# Small Object Detection in Traffic Scenes Based on Attention Feature Fusion

**DOI:** 10.3390/s21093031

**Published:** 2021-04-26

**Authors:** Jing Lian, Yuhang Yin, Linhui Li, Zhenghao Wang, Yafu Zhou

**Affiliations:** Faculty of Vehicle Engineering and Mechanics, School of Automotive Engineering, Dalian University of Technology, Dalian 116024, China; lianjing@dlut.edu.cn (J.L.); yinyuhang@mail.dlut.edu.cn (Y.Y.); zhwangv@mail.dlut.edu.cn (Z.W.); dlzyf@dlut.edu.cn (Y.Z.)

**Keywords:** traffic scenes, object detection, multi-scale channel attention, attention feature fusion

## Abstract

There are many small objects in traffic scenes, but due to their low resolution and limited information, their detection is still a challenge. Small object detection is very important for the understanding of traffic scene environments. To improve the detection accuracy of small objects in traffic scenes, we propose a small object detection method in traffic scenes based on attention feature fusion. First, a multi-scale channel attention block (MS-CAB) is designed, which uses local and global scales to aggregate the effective information of the feature maps. Based on this block, an attention feature fusion block (AFFB) is proposed, which can better integrate contextual information from different layers. Finally, the AFFB is used to replace the linear fusion module in the object detection network and obtain the final network structure. The experimental results show that, compared to the benchmark model YOLOv5s, this method has achieved a higher mean Average Precison (mAP) under the premise of ensuring real-time performance. It increases the mAP of all objects by 0.9 percentage points on the validation set of the traffic scene dataset BDD100K, and at the same time, increases the mAP of small objects by 3.5%.

## 1. Introduction

In traffic scenes, the visual perception technology of intelligent vehicles can help automatic driving systems to perceive complex environments accurately and in time, which is a requirement for avoiding collisions and for safe driving. With the rapid development of computer vision technology, vehicle visual perception is increasingly being adopted in the field of automatic driving. For example, object detection based on deep learning has played a very important role in the field of automatic driving.

Object detection involves the delineation of the bounding box of an object to be detected in the given image, and then the determination of the class that the object in the box belongs to. Due to their large amount of calculations, redundant marker boxes, and poor robustness of manual features, traditional object detection algorithms are currently being replaced by their deep learning counterparts. Lightweight real-time object detection models, such as the “you only look once” (YOLO) algorithm [1,2,3], the single shot multibox detector (SSD) algorithm [4], Light-Head R-CNN [5], and ThunderNet [6], have already demonstrated good detection effects in actual application scenarios.

At present, the prevailing deep learning-based object detection algorithms, such as YOLOv5 [7], treat each region of the whole feature map equally by default, that is, each region has the same contribution to the final detection result. This means that they do not weigh the convolution features extracted from the network according to their position and importance. However, compared with simple ordinary scenes, there are usually more complex and rich semantic features around the object to be detected in actual traffic scenes. If the features of the object area are weighted according to their importance, the objects to be detected can be better positioned in the feature map and the detection accuracy and generalization ability of the model can be improved.

Furthermore, in traffic scenes, there are many small objects in the distance. These objects offer limited feature information due to their relatively small size, which makes detection more difficult. Research on small object detection includes a deconvolutional single shot detector (DSSD) [8], scale normalization for image pyramids (SNIP) [9], high-resolution detection network (HRDNet) [10], etc. The DSSD algorithm mainly improves the detection performance of the object detector for small objects by using a better feature extraction network and adding context information. The SNIP algorithm uses a novel training scheme, called scale normalization for image pyramids (SNIP), which selectively back-propagates the gradients of object instances of different sizes as a function of the image scale to better detect small objects. The HRDNet algorithm feeds high-resolution input into a shallow network to reserve more positional information while feeding low-resolution input into a deep network to extract more semantics. By extracting various features from high to low resolutions, the algorithm improves the detection performance of small objects as well as maintaining the detection performance of medium and large objects. These algorithms each have their own advantages and limitations. Improving the detection of small objects in traffic scenes as much as possible is also one of the current research hotspots in the field of visual perception for autonomous vehicles. The YOLOv5 model is a milestone object detection method, which achieves a good balance between accuracy and speed, but it still has the possibility for improvement in small object detection problems in traffic scenes.

In response to the above problems, in this paper, we first propose an MS-CAB to alleviate the problems caused by scale changes to small object detection. This block effectively improves the feature inconsistency between objects at different scales, and at the same time, focuses attention on the objects in the area that need to be focused on, which reduces the unnecessary shallow feature information of the background. In other studies [11,12], the attention mechanism also considers the scale, such as by aggregating contextual information through convolution kernels of different sizes or from the feature pyramid inside the attention module. The MS-CAB proposed here aggregates contextual information along the channel dimensions of the feature map. It can not only focus on large objects that are distributed globally, but also deal with small objects that are distributed more locally. This block helps the model to detect and identify objects with extreme size differences.

Second, based on MS-CAB, an AFFB is proposed that is different from linear fusion schemes such as addition and concatenation, which are completely context-independent. The block is non-linear and can better capture the contextual information from different network layers by fusing features that are inconsistent semantically and in terms of scale. By replacing the simple addition or concatenation operation with the AFFB, a network model with fewer parameters and higher detection accuracy can be obtained, and the detection effect of small objects is improved greatly.

The remainder of this paper is organized as follows: Section 2 introduces the related works and existing problems of the three topics of object detection, attention mechanisms, and feature fusion. Section 3 briefly introduces the benchmark model, YOLOv5s, and then elaborates on the principle and structure of the proposed MS-CAB and the AFFB. Section 4 presents the experiments and an analysis of the results. The paper ends with our conclusions and suggestions for future work.

## 2. Related Works

### 2.1. Object Detection

Object detection algorithms are mainly divided into one-stage and two-stage methods. Relatively speaking, one-stage object detection algorithms have better real-time performance, but lower accuracy, while two-stage algorithms have better accuracy, but weaker real-time performance. He et al. proposed a two-stage spatial pyramid pooling network (SPPNet) in 2014 [13]. By introducing a spatial pyramid pooling layer, the convolutional neural network (CNN) can receive inputs of non-fixed size without considering the size of the region of interest. The SPPNet method was ultimately 20 times faster than R-CNN [14], with comparable accuracy. Ren et al. proposed Faster R-CNN [15], and the region proposal network (RPN) candidate box generation algorithm based on Fast R-CNN [16], which greatly improved the speed of object detection. Besides, Lin et al. proposed feature pyramid networks (FPN) [17], which solved the multi-scale problem in object detection. Through a relatively simple network connection change, the detection effect of small objects is greatly improved while maintaining the original model’s computational load. The YOLO algorithm [1], which divides the image into multiple regions, formulates the bounding box, and predicts the probability of an object belonging to a class at the same time, was proposed by Redmon et al. It was the first one-stage object detection algorithm based on deep learning and started a new approach towards object detection. The author subsequently proposed the improved versions of YOLOv2 [2] and YOLOv3 [3], which further improved the detection accuracy while maintaining a relatively high detection speed. Then, Liu et al. proposed the SSD algorithm [4], which greatly improved the accuracy of object detection by introducing multi-reference and multi-resolution detection technology, especially for small objects.

To solve the problem of imbalance between positive and negative categories, Lin et al. proposed the RetinaNet algorithm [18], in which the focal loss is derived so that the algorithm can maintain a relatively fast detection speed, while the detection accuracy can be equivalent to that of two-stage object detection algorithms. Zhu et al. proposed the feature selective anchor-free (FSAF) module [19], which can be inserted into a one-stage detector with a feature pyramid structure to enhance the decision feature layer to which each input instance belongs to make full use of the performance of FPN, and this method has a high mAP value and little additional computation. Zhou et al. proposed CenterNet [20], which uses the object center point predicted by the heatmap instead of the anchor mechanism to predict the object and uses a higher-resolution output feature map. This network has strong scalability and simple model design, and thus achieves good results in detection speed and accuracy. Tan et al. proposed EfficientDet [21], which is a weighted bi-directional feature pyramid network (BiFPN) and a composite scale expansion method to refresh the mAP of the MS COCO dataset. In the above works, the detection accuracy of the object detection algorithms was improved to varying degrees. However, it is more important to make full use of the effective information of the input features to improve the detection performance of the model, especially the detection accuracy of small objects in traffic scenes while keeping the number of model parameters and the real-time performance of the model basically unchanged.

### 2.2. Attention Mechanism

When facing the external environment, the human visual system can quickly identify useful information and ignore irrelevant information. This characteristic is gradually being considered by computer vision researchers. Deep learning’s attention mechanism first appeared as an imitation of the human visual attention mechanism [22]. Non-local neural networks were proposed by Wang et al. in one of the important works on attention mechanisms in the field of computer vision [23]. Non-local operations calculate the response at a position as a weighted sum of the features at all positions and establish remote dependencies through self-attention, and they can also be used as general modules for various tasks, which can lead to improvements in the model accuracy. The squeeze-and-excitation network (SENet) [24] proposed by Hu et al. was the first attention mechanism that focused on the channel level dependencies of the model, and could adaptively adjust the characteristic response value of each channel. This network won the ImageNet 2017 classification competition and has been recognized as an important advancement in the field. Woo et al. proposed the convolutional block attention module (CBAM) [25], which contains two modules of channel attention and spatial attention so that the model has better performance and interpretability and pays more attention to foreground objects. The selective kernel network (SKNet) was proposed by Li et al. [26], which utilizes a building block called a selection kernel unit that allows each neuron to adaptively adjust the size of the receptive field, depending on the scale of the input information. Experiments showed that SKNet achieved better detection accuracy through its relatively low model complexity.

Roy et al. proposed spatial and channel squeeze-and-excitation (scSE) [27] for semantic segmentation. They proposed three variants of the squeeze-and-excitation (SE) module, channel squeeze-and-excitation (cSE), spatial squeeze-and-excitation (sSE), and scSE, as improvements of the SE module. Experiments have shown that these modules can enhance useful features and suppress useless ones. Combining the advantages of non-local neural networks and SENet, Cao et al. proposed the global context network (GCNet) [28], which uses a relatively small amount of calculations to optimize the global context modeling capabilities. Huang et al. proposed the criss-cross network (CCNet) [29], which was also based on Non-local Neural Networks. Its special feature is the novel criss-cross attention module, which can obtain contextual information from remote dependencies in a more effective way. The dual attention network (DANet) was proposed by Fu et al. [30], which adds two attention modules to a dilated fully convolutional network to model semantic dependencies in the spatial and the channel dimensions. This model achieved excellent results on the semantic segmentation dataset, Cityscapes.

Most of the above-mentioned attention mechanisms use global channel attention mechanisms, which are more suitable for the detection of large objects with a more global distribution. However, the scale range of objects is very large in actual traffic scenes. If only the contextual information is extracted from the global range, the detection effect of the model is better for large objects with more distribution in the global range, but will be weaker for small objects with more distribution in the local range. Therefore, a simplified multi-scale channel attention block composed of local channel attention and global channel attention is needed to adaptively extract contextual object information to improve the detection effect of small objects.

### 2.3. Feature Fusion

In many object detection tasks, the fusion of features at multiple scales is an important way to improve detection performance. Low-level object features have high resolution and usually contain more location and detail information, but they lack semantic information and have more noise. High-level features have richer semantic information after the convolution operation, but their resolution is reduced, and the location and detail information are lacking. Efficient integration of low-level and high-level features is key to improving a model’s detection performance. Depending on the sequence of feature fusion and prediction, feature fusion can be divided into early fusion and late fusion methods. Early fusion fuses features of different layers first and then trains predictors on the fused features, such as the addition operation in ResNet [31] and the concatenation operation in U-Net [32]. Late fusion improves the detection performance by combining the detection results of different layers, and can be mainly divided into two types. The first separately predicts the features of multiple scales before fusion, and then the obtained prediction results are processed comprehensively, such as in SSD [4], multi-scale CNN [33], etc. The second approach uses the idea of feature pyramid networks for reference, and then predicts after fusing the features, such as in YOLOv3 [3], feature fusion single shot multibox detector (FSSD) [34], etc.

The feature fusion problem is currently a research hotspot in the field of object detection. Chaib et al. improved the effect of feature fusion using a discriminant correlation analysis-based feature fusion strategy [35], which incurred only a small computational cost. The FSSD was proposed by Li et al. [34], which includes a feature fusion module. The module first fuses the features of different layers through concatenation operations to obtain a larger-scale feature, and then a feature pyramid is constructed on this feature map. This significantly improves the detection accuracy of the SSD model, with only a slight speed reduction. Lim et al. proposed the SSD with feature fusion and attention (FA-SSD) [36], which includes a feature fusion module and an attention module. The results showed that the network improved the accuracy of object detection, especially the detection performance of small objects.

Pang et al. proposed Libra R-CNN [37], which integrates features from different layers to obtain more balanced semantic feature information. Compared with [15] and [18], the detection effect on the MS COCO dataset was significantly improved. Ghaisi et al. proposed the neural architecture search feature pyramid network (NAS-FPN) [38], which uses a neural architecture search algorithm to customize a feature pyramid network that merges features across a range. This approach produced significant improvements in many object detection networks. An adaptive spatial feature fusion (ASFF) strategy was proposed by Liu et al. [39], which combines features of different layers by learning weight parameters. Experimental results showed that this method was superior to concatenation and element-wise methods. In addition to the feature fusion using deep learning technology, Gao et al. analyzed the limitations of only using deep learning methods, and proposed a new fusion logic that can effectively combine the advantages of known knowledge used by a traditional method with the self-extracted features learned by a deep learning method [40]. A better detection performance can be achieved by properly designing traditional and deep learning detectors. However, the above methods of feature fusion are biased towards constructing complex paths to combine the features of different network layers or groups. They are all too complicated. Therefore, we propose an AFFB with a simple structure to improve the integration of various object context features in traffic scenes using fewer parameters and smaller models to ultimately improve the network’s object detection performance, especially the detection accuracy of small objects.

## 3. Benchmark Model and Proposed Methods

In this section, we briefly introduce the benchmark model YOLOv5s, then elaborate on the principle and structure of the proposed MS-CAB, and finally present the AFFB based on MS-CAB.

### 3.1. The YOLOv5s Benchmark Model

The development of the YOLO series ushered in a change in object detection technology through the adoption of deep learning. At present, the YOLO series includes YOLOv1 [1], YOLOv2 [2], YOLOv3 [3], YOLOv4 [41], and YOLOv5 [7]. The YOLOv5 model is the latest iteration of the model, and constitutes an improvement over YOLOv4. The model is faster, more accurate, has fewer model parameters, and can be more easily adapted to various devices embedded in vehicles. The YOLOv5 model refers to four models of different sizes, namely, YOLOv5s, YOLOv5m, YOLOv5l, and YOLOv5x, where smaller models have fewer parameters, lower accuracy, and are faster. To better meet the real-time requirements of object detection in traffic scenes, in this study, we chose the YOLOv5s model as the benchmark model for improvement.

### 3.2. Multi-Scale Channel Attention Block

**Figure 1:**
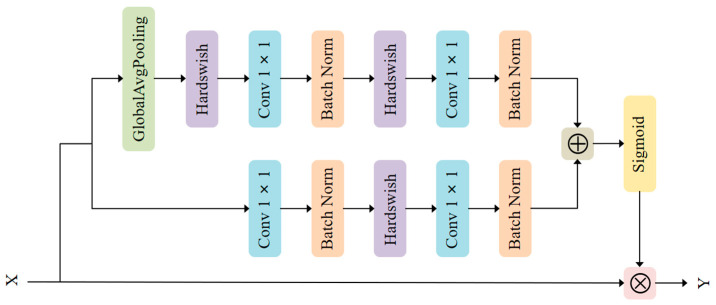
The MS-CAB structure. The global average pooling branch is the global channel attention, while the other is the local channel attention.

Based on the idea of combining local and global features in the convolutional neural networks adopted in ParseNet [42] and multi-scale channel attention [43], we propose MS-CAB, with the main difference being that we use 1×1 convolution rather than kernels of different sizes to control the channel attention scale. Similar to spatial attention, channel attention also has a scale, and the variable that controls that scale is the size of the pooling. Figure 1 shows a diagram of the MS-CAB structure, which is divided into two scales, the local scale and the global scale, where context features are aggregated through both scales. The branch that uses global average pooling is the global scale, while the other is the local scale. This block gathers contextual information along the channel dimension of the feature map, and can simultaneously focus on large objects that are more distributed in the global range and small objects that are distributed more in the local range, which helps the model to detect and identify objects with extreme scale changes in traffic scenes. In the following, we introduce the details of the implementation of the proposed MS-CAB.

Suppose that the output of a certain layer in the middle of the network is X and $X\in R^{C\times H\times W}$, where C is the channel number of the feature map, and H and W are the height and width of the feature map, respectively. Then, X is used as the input of MS-CAB. The global and local channel attention can be obtained by changing the pooling size, and 1×1 convolution is used as the local channel context aggregator to extract the channel interaction at each spatial location. The local channel context $L(X)\in R^{C\times H\times W}$ can be expressed as

(1) $$L(X)=BN(Conv_{2}(Hs(BN(Conv_{1}(X))))),$$

where the convolution kernel parameters of $Conv_{1}$ and $Conv_{2}$ are $\frac{C}{r}\times C\times 1\times 1$ and $C\times \frac{C}{r}\times 1\times 1$, r is the channel reduction ratio, BN stands for batch normalization [44], and Hs stands for the Hardswish activation function [45]. The local channel context L(X) has the same shape as the input feature map X, and retains and highlights the richly detailed information of the low-level features. It focuses more on the small object information present in the local range.

The global channel context $G(X)\in R^{C\times 1\times 1}$ can be expressed as

(2) $$G(X)=BN(Conv_{2}(Hs(BN(Conv_{1}(Hs(g(X))))))),$$

(3) $$g(X)=\frac{1}{H\times W}\sum_{i=1}^{H}\sum_{j=1}^{W}X_{\left[:,i,j\right]}$$

where $g(X)\in R^{C}$ stands for global average pooling. Here, G(X) has the same number of channels as the input feature map X and pays more attention to large object information that is distributed more globally.

Combining the local channel context L(X) and the global channel context G(X), the output $Y\in R^{C\times H\times W}$ of the MS-CAB can be expressed as follows:

(4) Y=X⊗MSCAB(X)=X⊗σ(L(X)⊕G(X))

where $MSCAB(X)\in R^{C\times H\times W}$ represents the output weight of the MS-CAB, σ represents the sigmoid function, ⊗ represents element-wise multiplication, and ⊕ represents the addition of the broadcast mechanism.

**Figure 2:**
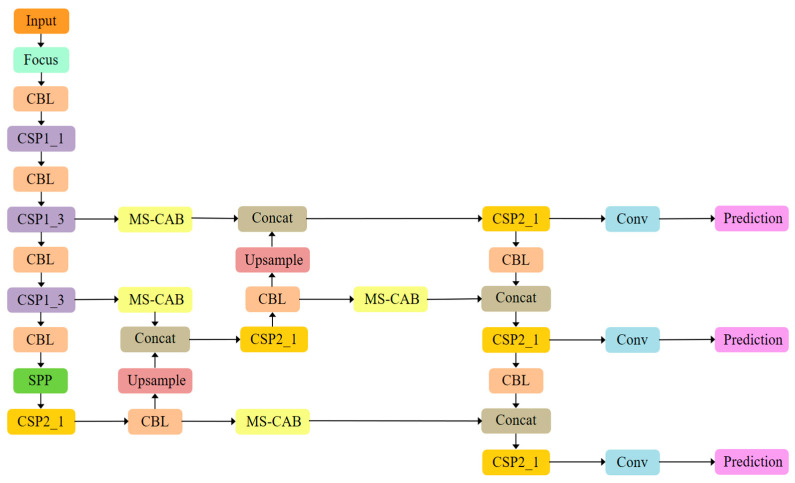
The MS-CAB_YOLOv5s network structure.

**Figure 3:**
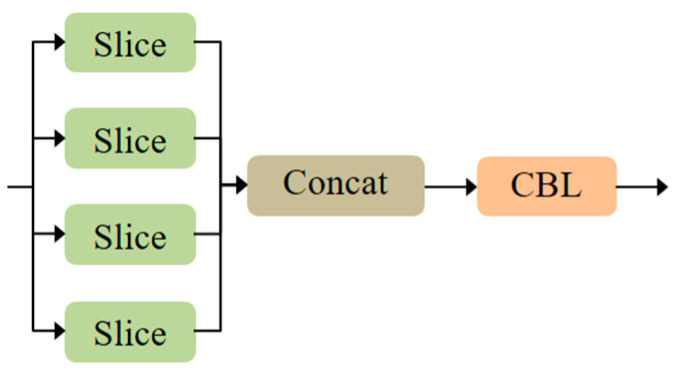
Composition of the “Focus” block.

**Figure 4:**
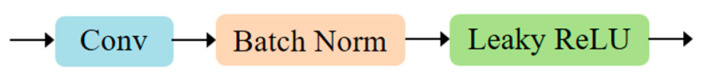
Composition of the CBL block.

**Figure 5:**
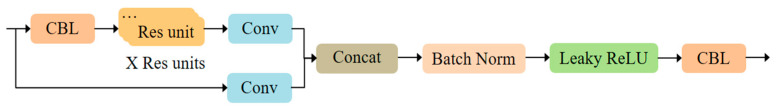
Composition of the CSP1_X block.

**Figure 6:**
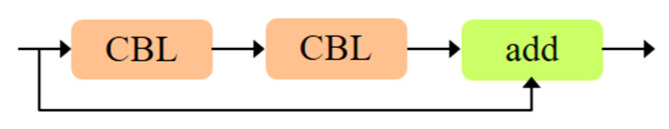
Composition of the residual unit block.

**Figure 7:**
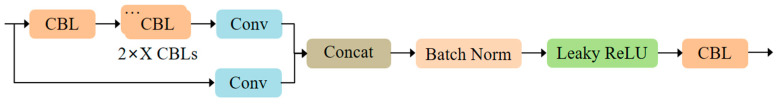
Composition of the CSP2_X block.

**Figure 8:**
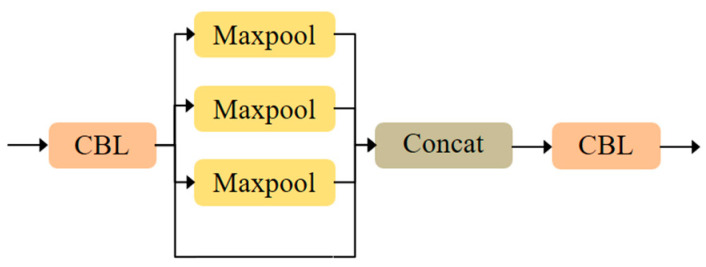
Composition of the SPP block.

The proposed MS-CAB was embedded in the four Concat operation branches of the YOLOv5s model, and a new network model, MS-CAB_YOLOv5s, was obtained. The network structure diagram is shown in Figure 2. In the diagram, “Input” refers to the network input, and “Prediction” is the prediction result made by the network on the feature map on three scales. “Upsample” represents an upsampling operation, “Concat” denotes a concatenation operation, and “Conv” denotes a convolution operation. The composition of the “Focus” block is shown in Figure 3. It performs a slicing operation on the input red/green/blue (RGB) image, ultimately integrating the width and height information into the channel dimension. Its main function is to reduce floating point operations and improve the running speed of the model. The CBL block is composed of a convolution layer, batch normalization, and the Hardswish activation function, and its composition is shown in Figure 4. The YOLOv5s model contains two cross stage partial (CSP) structures [46], of which the CSP1 structure is used in the backbone of the network, while the CSP2 structure is used in the neck of the network. The composition of CSP1_X is shown in Figure 5. Here, CSP1_X indicates that it contains X residual units; for example, CSP1_1 contains one residual unit, and CSP1_3 contains three residual units. The composition of each residual unit is shown in Figure 6. The composition of CSP2_X is shown in Figure 7. Here, CSP2_X means that, in addition to the first CBL component, there are 2×X CBL components in the middle. The size of the convolution kernel in the first CBL component is 1×1, while in the second CBL component it is 3×3. For example, in addition to the first CBL component in CSP2_1, there are 2×1=2 CBL components in the middle, and the convolution kernel sizes in the two CBL components are 1×1 and 3×3, respectively. The SPP block uses the maximum pooling method to perform “Concat” operations on feature maps of different scales, and its composition is shown in Figure 8.

### 3.3. Attention Feature Fusion Block

**Figure 9:**
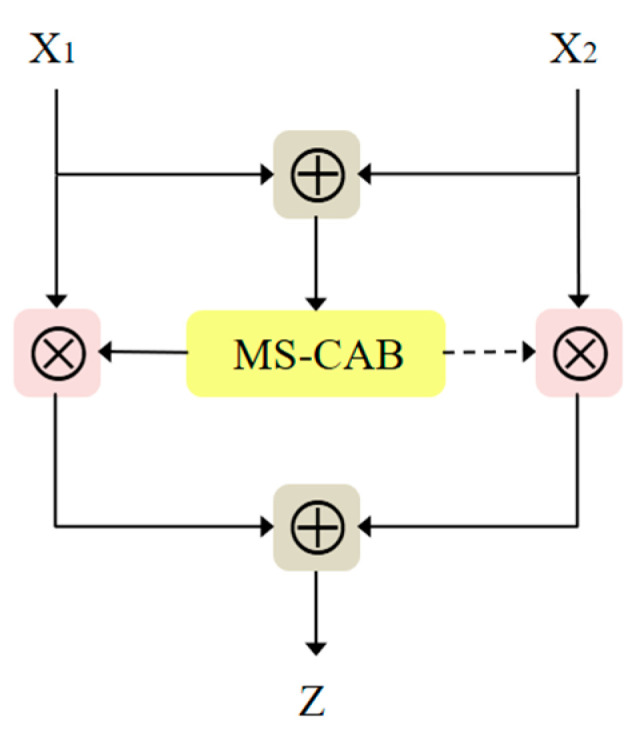
The AFFB structure.

In combination with the multi-scale channel attention block proposed above, we propose AFFB, which can better capture contextual information from different network layers by fusing semantic and scale-inconsistent features and thus achieve better object detection. Figure 9 is a structure diagram of the AFFB. Due to the presence of the multi-scale channel attention block, the output $Z\in R^{C\times H\times W}$ of the AFFB can be expressed as

(5) $$Z=MSCAB(X_{1}⊕X_{2})⊗X_{1}+(1-MSCAB(X_{1}⊕X_{2}))⊗X_{2}$$

where $X_{1}\in R^{C\times H\times W}$ and $X_{2}\in R^{C\times H\times W}$ are two input feature maps, with $X_{1}$ being a low-level semantic feature map and $X_{2}$ a high-level semantic feature map. The values of the fusion weights $MSCAB(X_{1}⊕X_{2})$ and $1-MSCAB(X_{1}⊕X_{2})$ are both between 0 and 1, which corresponds to a weighted averaging operation between $X_{1}$ and $X_{2}$.

**Figure 10:**
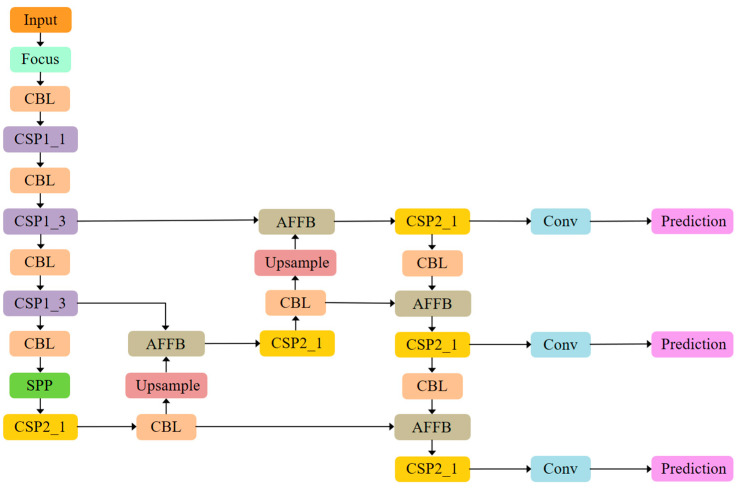
The AFFB_YOLOv5s network structure.

In YOLOv5s, linear feature fusion is performed through concatenation, which only yields a fixed linear aggregation of feature maps, and is not adaptable to the object to be detected. The AFFB has fewer parameters, is non-linear, and can capture the contextual information from different network layers better through the fusion of features that are inconsistent semantically and in terms of scale. The four “Concat” operations are then replaced in the YOLOv5s model with the proposed AFFB to obtain a new network model AFFB_YOLOv5s, as shown in Figure 10.

## 4. Experiments and Result Analysis

### 4.1. Datasets and Experimental Settings

#### 4.1.1. Datasets

In this paper, the object detection task is oriented towards traffic scenes, and thus the experimental part mainly used the BDD100K dataset [47], while the PASCAL VOC dataset [48] was used as an auxiliary validation dataset.

The BDD100K dataset is the largest open autonomous driving dataset, and includes ten categories of traffic scene objects: car, bus, person, bike, truck, motor, train, rider, traffic sign, and traffic light. It has a very rich diversity of geography, environments, and weather to enable models to recognize a variety of complex traffic scenes and make the models’ generalization ability stronger at the same time. The dataset has a total of 100,000 images with a resolution of 1280 × 720 pixels. The official usage guidelines recommend splitting the dataset into a training set, a validation set, and a test set at a 7:1:2 ratio. As the labels of the test set are not disclosed, we used the validation set to test the model and evaluate the model’s detection performance of the model. The final training set consisted of 70,000 images, and the test set consisted of 10,000 images. (The BDD100K dataset is available at https://bdd-data.berkeley.edu, accessed on 25 November 2020).

The PASCAL VOC dataset is a commonly used object detection dataset, and it includes two parts, VOC2007 and VOC2012, with a total of 20 categories: airplane, bicycle, bird, boat, bottle, bus, car, cat, chair, cow, dining table, dog, horse, motorbike, person, potted plant, sheep, sofa, train, and TV monitor. In this paper, 22,136 images of the VOC2007 and VOC2012 training and validation sets were used for model training. The test set of VOC2007 has a total of 4952 images and was used to evaluate the detection performance of the model. (The PASCAL VOC dataset is available at http://host.robots.ox.ac.uk/pascal/VOC/, accessed on 30 November 2020).

#### 4.1.2. Experimental Settings

(a) Network loss function

The loss function of the network designed in this paper is divided into three parts: bounding box regression loss $L_{box}$, confidence loss $L_{obj}$, and classification loss $L_{cls}$. The total loss of the network is the sum of the three functions. The bounding box regression loss uses the complete intersection over union (CIoU) loss [49], and both the confidence loss and classification loss use the binary cross-entropy (BCE) with logits loss (BCEWithLogitsLoss). The CIoU loss considers three important geometric factors of the bounding box regression loss: the overlap area between the prediction and the ground truth boxes; the center point distance of the prediction and the ground truth boxes; and the aspect ratio between the prediction and the ground truth boxes, which improves the speed and accuracy of bounding box regression. The bounding box regression loss $L_{box}$ can be expressed as follows:

(6) $$L_{\mathrm{box}}=1-CIoU=1-(IoU-\frac{\rho^{2}}{c^{2}}-\alpha v)$$

where intersection-over-union (IoU) is the ratio of the intersection area to the union area of the prediction box and the ground truth box, ρ is the Euclidean distance between the center points of the prediction and the ground truth boxes, and c is the diagonal length of the smallest enclosing box covering both the prediction box and the ground truth box. Besides, α is the trade-off parameter, which is defined as

(7) $$\alpha =\frac{v}{(1-IoU)+v}$$

here, v is a parameter that measures the consistency of the aspect ratio between the ground truth box and the prediction box, and it is expressed as follows:

(8) $$v=\frac{4}{\pi^{2}}{\left(arctan\frac{w^{gt}}{h^{gt}}-arctan\frac{w^{p}}{h^{p}}\right)}^{2}$$

where $w^{gt}$ and $h^{gt}$ are the width and height of the ground truth box, while $w^{p}$ and $h^{p}$ are the corresponding values of the prediction box.

The BCEWithLogitsLoss mainly measures the binary cross-entropy between the target value and the output value of the model. It can be expressed as

(9) $$L_{n}=-w_{n}[y_{n}log\sigma (x_{n})+(1-y_{n})log(1-\sigma (x_{n}))]$$

where $w_{n}$ is the loss weight of each category, $y_{n}$ is the target value, $x_{n}$ is the output value of the model, and σ is the sigmoid function.

(b) Training parameter settings

In this study, we used the stochastic gradient descent algorithm [50] to optimize the loss function. The momentum was set to 0.937, the weight decay coefficient was set to 0.0005, and the initial learning rate was set to 0.01. We used warmup training [51], cosine annealing [52], gradient accumulation, exponential moving average, and other optimization strategies. In terms of data augmentation, in addition to the most advanced mosaic data augmentation method [41], common data augmentation methods, such as random hue, saturation, value transformation, image horizontal and vertical translation, image scaling, and image left and right flip, were also used. The batch size was set to 32, the epochs were set to 300, and the resolution size of the input image was set to 640×640. The channel reduction ratio r was set to 4. The *k*-means clustering algorithm was used to obtain new anchor boxes. Other parameter settings were consistent with the default settings of YOLOv5. The computer configuration used in the experiment is shown in Table 1.

(c) Testing parameter settings

The batch size was set to 1, the resolution size of the input image was set to 640×640, the confidence threshold for the filtering prediction box was set to 0.001, and the IoU threshold for non-maximum suppression was set to 0.6. Other parameter settings were consistent with the default YOLOv5 settings.

### 4.2. Quantitative Result Analysis

The three models, YOLOv5s, MS-CAB_YOLOv5s, and AFFB_YOLOv5s, were trained on the BDD100K dataset to test the effectiveness of the proposed MS-CAB and AFFB blocks. Five indicators commonly used in the field of object detection, namely, precision, recall, mAP, frames per second (FPS), and the number of parameters, were used to quantitatively evaluate the accuracy of the model [7]. To quantitatively study the impact of the proposed improvements on the detection of small objects, we examined small objects of the size defined by the COCO dataset [53], that is, those with a pixel area smaller than 32×32 pixels. Moreover, to verify the generalization ability of the model on other datasets, we used the same parameter settings as above on the public dataset PASCAL VOC for network training, and then tested to complete the auxiliary validation.

The accuracy evaluation results of the three models on the BDD100K validation set are shown in Table 2. It is evident that under the premise of ensuring the real-time requirements of a vehicle’s environment perception, compared with the original YOLOv5s model, the precision, recall, and mAP of the MS-CAB_YOLOv5s and AFFB_YOLOv5s models proposed in this paper were improved to varying degrees. Among them, the mAP of the AFFB_YOLOv5s model increased by 0.9 percentage points, which is a significant improvement given the complexity of the BDD100K traffic scene dataset. The 63 FPS achieved by both improved networks can fully meet the real-time requirements of vehicles’ environment perception systems. Furthermore, the parameters of the model were reduced to a certain extent. The size of the model is only 14.7 MB, which makes it quite suitable for embedded vehicle platforms.

The BDD100K dataset is a traffic scene dataset, and thus contains many cars and traffic signs at a distance with a pixel area less than 32×32 pixels. These objects are defined as small objects that need to be detected. Table 3 shows the comparison results of the three models for small object detection performance. Compared with the original YOLOv5s model, the MS-CAB_YOLOv5s and AFFB_YOLOv5s models proposed in this paper had a significantly improved precision of small object detection, while the recall decreased slightly, and the mAP, respectively, improved by 1.6 and 3.5 percentage points. This shows that the MS-CAB and AFFB significantly improved the model’s detection effect on small objects.

To verify the generalization ability of the model, the three models were trained and tested on the PASCAL VOC dataset. The performance comparison for each model is shown in Table 4. Under the premise of ensuring real-time performance, the two models, MS-CAB_YOLOv5s and AFFB_YOLOv5s, had improved precision, recall, and mAP. This again verifies the effectiveness of the MS-CAB and AFFB to improve the performance of object detection. At the same time, it shows that our improved model can adapt to different datasets or scenes and has good generalization ability.

### 4.3. Comparative Analysis of Detection Results

**Figure 11:**
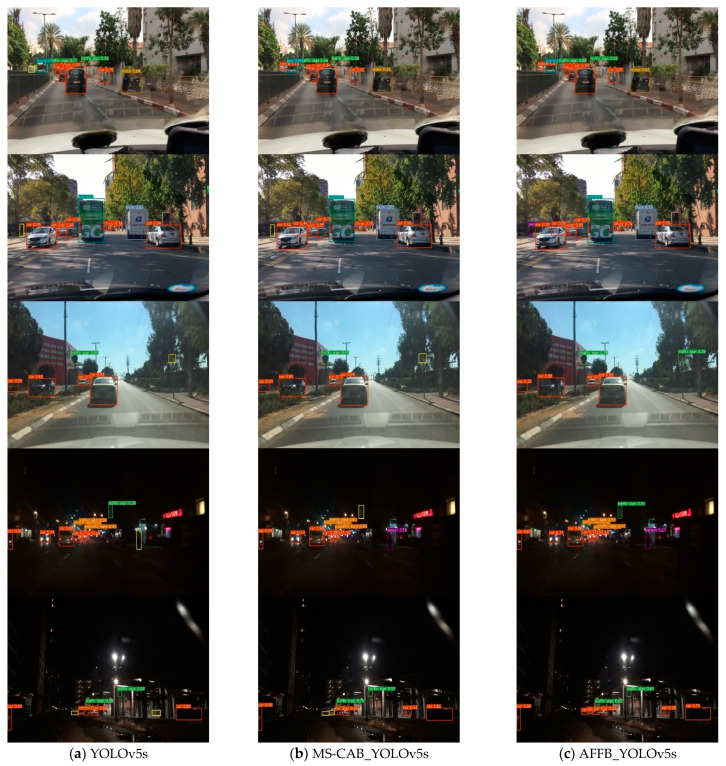
Comparison of the detection results of YOLOv5s, MS-CAB_YOLOv5s, and AFFB_YOLOv5s.

Figure 11 shows a visual comparison of the detection results of the YOLOv5s model, the MS-CAB_YOLOv5s model, and the AFFB_YOLOv5s model. To see the differences between the three models more easily, the yellow rectangles in the detection result of column (a) in Figure 11 indicate the objects that were not detected by YOLOv5s. Similarly, the yellow rectangles in the detection result of column (b) indicate the objects that were not detected by MS-CAB_YOLOv5s. The AFFB_YOLOv5s model could detect small objects with small pixel areas, such as cars, people, and traffic signs, at long distances that were not detected by the YOLOv5s model. At the same time, the detection effect was also excellent under dark night conditions. Moreover, compared with the benchmark model YOLOv5s, the detection effect of the MS-CAB_YOLOv5s model was better. It could detect some objects that the YOLOv5s model did not detect, but its effect was not as good as that of AFFB_YOLOv5s. For example, in column (b) of Figure 11, the person on the left side of the figure on the second row and the traffic sign on the right side of the figure on the third row were not detected by the MS-CAB_YOLOv5s model, but they were all accurately detected by the AFFB_YOLOv5s model. Based on these detection results in Figure 11, both the MS-CAB_YOLOv5s model and the AFFB_YOLOv5s model could improve the effect of object detection in traffic scenes, and the AFFB_YOLOv5s model had the best detection effect, especially for small objects that are away from the vehicle, which is of great significance for improving the stability and efficiency of automatic driving systems and preventing traffic accidents.

## 5. Conclusions and Future Work

The high accuracy and fast real-time performance of object detection algorithms are very important for the safety and real-time control of autonomous vehicles. In this paper, we presented a small object detection method for traffic scenes based on attention feature fusion for autonomous driving systems as an improvement to the YOLOv5s architecture. To aggregate the effective information at the local and global scales, MS-CAB simultaneously focuses on small objects that are more distributed within a local range and large objects that are more distributed on the global range. Using AFFB to fuse contextual information from different network layers, we obtain a model with fewer parameters and higher accuracy. Under the condition of meeting the real-time requirements of vehicles’ environment perception systems, compared with the benchmark model YOLOv5s, the model proposed in this paper increased the mAP of all objects on the validation set of the traffic scene dataset BDD100K by 0.9 percentage points. Specifically, small objects’ mAP was increased by 3.5%. Therefore, the model achieves a better balance between object detection accuracy and speed in traffic scenes, and can effectively improve the performance of vision-based object detection systems for autonomous vehicles.

Since our proposed method is essentially based on deep learning, there are some general limitations. First, the interpretability of deep learning is poor. It learns the implicit relationship between input and output features, but not the causal relationship. Secondly, the neural network has many parameters, and network training requires a large amount of time and relatively large computing power. Therefore, the deep learning method requires stronger computer hardware equipment. Finally, the accuracy of the model based on the deep learning method greatly relies on the collected data, and the accuracy of the dataset label directly determines the accuracy of the model detection. A traditional method based on manual feature extraction is a beneficial supplement to the deep learning method. In future research, we will try to combine the two methods to further improve object detection performance. We plan to deploy the model proposed in this paper to embedded vehicle devices to develop more convenient portable applications. Moreover, we will explore the extent to which the proposed blocks improve the performance of larger YOLOv5 models.

## Figures and Tables

**Table 1:** Computer configuration.

Project	Content
CPU	Intel Xeon E5-2620 v4
RAM	32GB
GPU	NVIDIA TITAN Xp
Operating System	Ubuntu 18.04.5 LTS
Cuda	Cuda 10.1 with Cudnn 7.5.1
Data Processing	Python 3.8, OpenCV
Deep Learning Framework	Pytorch 1.7.0

**Table 2:** Model performance comparison on the BDD100K validation set.

Model	Precision (%)	Recall (%)	mAP (%)	FPS	Parameters (M)
YOLOv5s	32.5	57.7	50.6	77	7.28
MS-CAB_YOLOv5s	32.5	58.1	51.0	63	7.45
AFFB_YOLOv5s	33.0	58.3	51.5	63	7.20

**Table 3:** Comparison of models on small object detection performance.

Model	Precision (%)	Recall (%)	mAP (%)
YOLOv5s	11.7	51.9	21.5
MS-CAB_YOLOv5s	16.4	49.8	23.1
AFFB_YOLOv5s	23.1	48.6	25.0

**Table 4:** Performance comparison of models on PASCAL VOC test set.

Model	Precision (%)	Recall (%)	mAP (%)	FPS	Parameters (M)
YOLOv5s	60.3	82.3	79.4	76	7.31
MS-CAB_YOLOv5s	62.0	82.7	80.2	61	7.48
AFFB_YOLOv5s	63.4	82.9	80.8	61	7.23

## Data Availability

Not applicable.
